# Dramatic increase of third-generation cephalosporin-resistant *E. coli *in German intensive care units: secular trends in antibiotic drug use and bacterial resistance, 2001 to 2008

**DOI:** 10.1186/cc9062

**Published:** 2010-06-14

**Authors:** Elisabeth Meyer, Frank Schwab, Barbara Schroeren-Boersch, Petra Gastmeier

**Affiliations:** 1 Institute of Hygiene and Environmental Medicine, Charité-University Medicine Berlin, Hindenburgdamm 27, 12203 Berlin, Germany; 2 National Reference Centre for the Surveillance of Nosocomial Infections, Hindenburgdamm 27, 12203 Berlin, Germany; 3 Institute of Environmental Health Sciences, University Medical Center Freiburg, Breisachstraße 115B, 79106 Freiburg, Germany

## Abstract

**Introduction:**

The objective of the present study was to analyse secular trends in antibiotic consumption and resistance data from a network of 53 intensive care units (ICUs).

**Methods:**

The study involved prospective unit and laboratory-based surveillance in 53 German ICUs from 2001 through 2008. Data were calculated on the basis of proportions of nonduplicate resistant isolates, resistance densities (that is, the number of resistant isolates of a species per 1,000 patient-days) and an antimicrobial usage density (AD) expressed as daily defined doses (DDD) and normalised per 1,000 patient-days.

**Results:**

Total mean antibiotic use remained stable over time and amounted to 1,172 DDD/1,000 patient-days (range 531 to 2,471). Carbapenem use almost doubled to an AD of 151 in 2008. Significant increases were also calculated for quinolone (AD of 163 in 2008) and third-generation and fourth-generation cephalosporin use (AD of 117 in 2008). Aminoglycoside consumption decreased substantially (AD of 86 in 2001 and 24 in 2008). Resistance proportions were as follows in 2001 and 2008, respectively: methicillin-resistant *Staphylococcus aureus *(MRSA) 26% and 20% (*P *= 0.006; trend test showed a significant decrease), vancomycin-resistant enterococcus (VRE) *faecium *2.3% and 8.2% (*P *= 0.008), third-generation cephalosporin (3GC)-resistant *Escherichia*. *coli *1.2% and 19.7% (*P *< 0.001), 3GC-resistant *Klebsiella pneumoniae *3.8% and 25.5% (*P *< 0.001), imipenem-resistant *Acinetobacter baumannii *1.1% and 4.5% (*P *= 0.002), and imipenem-resistant *K. pneumoniae *0.4% and 1.1%. The resistance densities did not change for MRSA but increased significantly for VRE *faecium *and 3GC-resistant *E. coli *and *K. pneumoniae*. In 2008, the resistance density for MRSA was 3.73, 0.48 for VRE, 1.39 for 3GC-resistant *E. coli *and 0.82 for *K. pneumoniae*.

**Conclusions:**

Although total antibiotic use did not change over time in German ICUs, carbapenem use doubled. This is probably due to the rise in 3GC-resistant *E. coli *and *K. pneumoniae*. Increased carbapenem consumption was associated with carbapenem-resistant *K. pneumoniae *carbapenemase-producing bacteria and imipenem-resistant *A. baumannii*.

## Introduction

In recent years, an increased effort has been directed towards controlling antibiotic use and raising public awareness of the need for prudent use of antibiotics [1]. There are two main reasons for this. The first is ecological, in that antibiotics induce and select for bacterial resistance [2]. Resistance is meanwhile considered a global threat, and pathogens susceptible to antibiotics are considered a common good [3]. Antimicrobial drug effectiveness cannot be taken for granted and antimicrobials are increasingly attaining the status of nonrenewable resources. The second reason is economic, in that antibiotics account for a large portion of a hospital's pharmacy budget, and in the face of restricted financial resources are therefore a main target for cost savings [4]. Over the past decade, many surveillance efforts have drawn attention to this phenomenon [5-8].

The Surveillance System of Antibiotic Use and Bacterial Resistance in Intensive Care Units (SARI) is an ongoing project, launched in 2000 and initially funded by the German Government, that collects data from its network of intensive care units (ICUs) [9-12]. SARI focuses on the critically ill because antimicrobial use in ICUs is among the highest in the hospital setting and consumption often runs in parallel to the pattern seen for resistance.

The goal of the present study is to give an overview of changes in antibiotic consumption and resistance in a network of ICUs over a period of 8 years (2001 through 2008).

## Materials and methods

SARI started in February 2000. Following a pilot phase, we first analysed data at the beginning of 2001. Data from 53 SARI ICUs were included in the analysis presented here: 21 of the 53 ICUs were interdisciplinary, 18 were surgical (of which four were neurosurgical) and 14 were medical. Most ICUs were located in hospitals affiliated with a university hospital (n = 30) or in university hospitals (n = 19). The median of hospital size was 790 beds (interquartile range 463 to 1,119 beds), and the median number of ICU beds was 12 (interquartile range 10 to 18 beds).

Data are fed back to the participants every 6 months. From 2001 through 2008, the numbers of ICUs reporting data to the project were 36, 35, 38, 40, 44, 46, 45 and 45, respectively. Forty-seven per cent of the ICUs sent data from all 8 years.

### Data collection

Monthly data on antimicrobial use were obtained from the computerised pharmacy databases. Consumption - that is, the antimicrobial usage density (AD) - was expressed as daily defined doses (DDD) and was normalised per 1,000 patient-days. The DDD are the standard adult daily dose of an antimicrobial agent for a 1-day treatment defined by the World Health Organisation (WHO ATC/DDD Index 2008) [13].

The ICUs indicated the number of isolates tested per month belonging to the following 13 sentinel bacterial species: *Staphylococcus aureus*, coagulase-negative staphylococci, *Enterococcus faecalis*, *Enterococcus faecium*, *Pseudomonas aeruginosa*, *Enterobacter cloacae*, *Citrobacter *spp., *Serratia marcescens*, *Acinetobacter baumannii*, *Stenotrophomonas maltophilia, Streptococcus pneumoniae, Escherichia coli *and *Klebsiella pneumoniae*. The susceptibility data were collected from the microbiology laboratory for these 13 pathogens regardless of whether they were associated with hospital-acquired or community-acquired infection or colonisation, or whether they were from clinical or surveillance cultures. Pathogens were specified as resistant by the clinical laboratory using interpretive criteria recommended by the German Industrial standard or CLSI. Copy strains - defined as an isolate of the same species showing the same susceptibility pattern throughout a period of 1 month in the same patient, no matter what the site of isolation - were excluded. Thirty-seven per cent of the SARI ICUs - 13 out of 35 SARI ICUs responded to a questionnaire on methicillin-resistant *S. aureus *(MRSA) management in 2008 - screened all patients for MRSA at admission. Questions on extended-spectrum β-lactamase (ESBL) screening were not included.

All data were anonymous and were collected in accordance with the German recommendations of good epidemiological praxis with respect to data protection [14]. As a federal law, the German Protection against Infection Act (Infektionsschutzgesetz §23) regulates the prevention and management of infectious disease in humans. All hospitals are obliged to collect and analyse continuously nosocomial infections and resistant pathogens [15]. These routine data were reported to the National Reference Centre of the Surveillance of Nosocomial Infections. Ethical approval and informed consent were thus not required.

### Statistical analysis

The proportion of resistant isolates was calculated by dividing the number of resistant isolates by the total number of the isolates of the same species tested against the corresponding antibiotic multiplied by 100. The incidence density of resistant isolates (RD) was defined as being the number of resistant isolates per 1,000 patient-days. Differences in consumption and resistance by type of ICU were tested using the Kruskal-Wallis test. From 2001 through 2008, trends in resistance were analysed by regression analysis using aggregated 3-monthly data (24 time points) and trends in antibiotic use were analysed using monthly data (96 time points). We tested whether the linear regression coefficient was significantly different from zero.

The significance level was *P *< 0.05 and all analyses were performed using EpiInfo 6.04 and SAS 9.2 (SAS Institute Inc., Cary, NC, USA).

## Results

From 2001 through 2008, a total of 53 ICUs from 30 hospitals reported data to SARI covering 1,335,855 patient-days. The mean length of stay was 4.2 days in 2001 and 4.0 days in 2008. The rate of ventilated patients ranged between 45 and 49% over the 8-year period. Altogether, 121,548 pathogens (53% of them Gram-positive) were isolated with a mean number of 91 pathogens per 1,000 patient-days.

**Figure 1:**
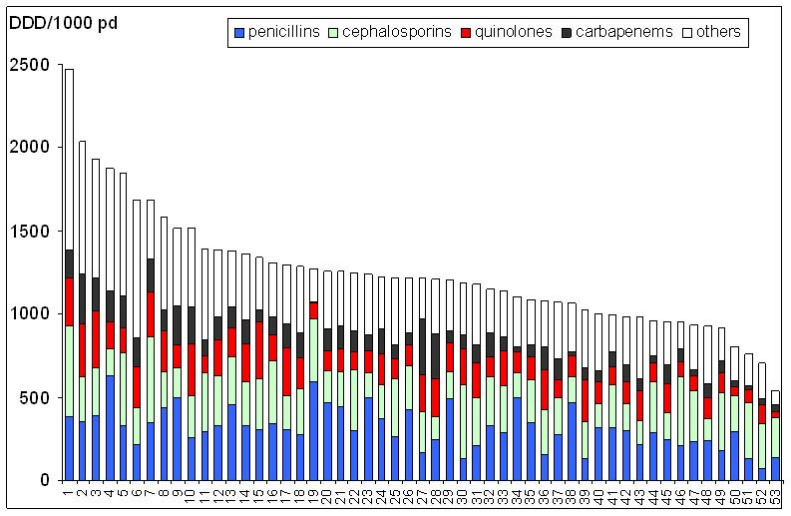
**Antibiotic consumption in 53 German intensive care units from 2001 to 2008**. DDD, defined daily doses; pd, patient-days.

Pooled mean antibiotic use over the 8-year period was 1,172 DDD/1,000 patient-days; that is, each patient on average received 1.2 DDD. Antibiotic consumption ranged from 531 to 2,471 DDD/1,000 patient-days (median 1,213 DDD/1,000 patient-days). The proportions of β-lactamase-sensitive penicillins, of penicillins with extended spectrum, of β-lactamase-resistant penicillins and of penicillins with β-lactamase inhibitor among the whole class of penicillins were 7.4%, 32.8%, 8.9%, and 51.9%, respectively (2001 to 2008). Figure 1 shows the heterogeneity of total antibiotic consumption and distribution over antibiotic classes in individual ICUs.

Total mean antibiotic use (without sulbactam) remained stable over time (*P *= 0.707). The AD was 1,180 DDD/1,000 patient-days in 2001 and was 1,167 DDD/1,000 patient-days in 2008. There was no difference either in total antibiotic use or in the trend over time by type of ICU.

**Figure 2:**
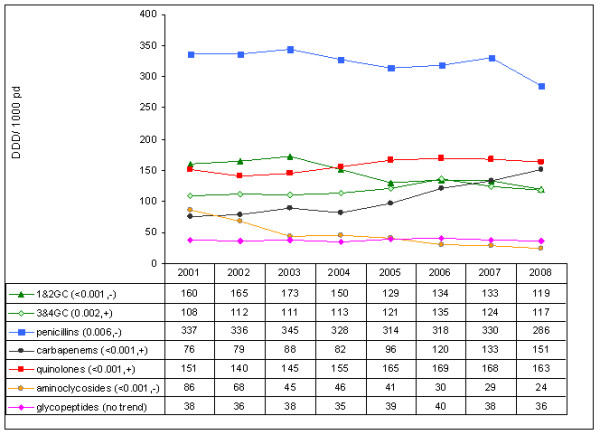
**Change in antibiotic consumption in German intensive care units from 2001 to 2008**. DDD, defined daily doses; pd, patient-days; 1&2GC, first-generation and second-generation cephalosporins; 3&4GC, third-generation and fourth-generation cephalosporins. *P *value for the linear regression coefficient and trend (increase, +; decrease, -) in parentheses.

Within the antibiotic classes, carbapenem use almost doubled to an AD of 151 in 2008 (Figure 2). Carbapenem use and third-generation cephalosporin (3GC) resistance correlated significantly (*P *= 0.036; correlation coefficient = 0.291). Significant increases were also calculated for quinolones and for third-generation and fourth-generation cephalosporin use. Consumption of the whole class of cephalosporins and penicillins, however, decreased significantly. Aminoglycosides showed the biggest decrease over time (*P *< 0.001). Macrolide use was significantly higher in medical ICUs, whereas second-generation cephalosporin consumption was significantly lower in medical ICUs than in surgical or interdisciplinary ICUs.

The most striking result was the continuous increase of 3GC-resistant *E. coli *(%), which equated to an almost 10-fold increase within just 8 years. Vancomycin-resistant *Enterococcus *(VRE) *faecium *quadrupled between 2006 and 2008 (Table 1). In contrast, the proportion of MRSA even decreased and the resistance proportions of imipenem-resistant or ciprofloxacin-resistant *P. aeruginosa *revealed no trend at all.

**Table 1:** Pooled mean antimicrobial resistance of selected pathogens in German intensive care units (number of ICUs), 2001 to 2008

Resistant pathogen (number tested against each antimicrobial)	2001 (n = 36)	2002 (n = 35)	2003 (n = 38)	2004 (n = 40)	2005 (n = 44)	2006 (n = 46)	2007 (n = 45)	2008 (n = 45)	*P *value*
Methicillin-resistant *Staphylococcus aureus *(27,446)	26.0	22.4	20.9	19.5	22.6	22.2	20.6	19.5	0.006 (-)
Vancomycin-resistant *Enterococcus faecium *(6,331)	2.3	1.2	1.2	5.4	5.4	2.2	3.6	8.2	0.008 (+)
3GC-resistant *Escherichia coli *(18,425)	1.2	1.8	2.9	3.8	3.7	5.1	11.1	10.5	< 0.001 (+)
Ciprofloxacin-resistant *Escherichia coli *(16,184)	8.3	11.9	14.1	16.5	18.2	16.4	20.9	24.2	< 0.001 (+)
3GC-resistant *Klebsiella pneumoniae *(7,457)	3.8	12.2	5.9	6.5	6.5	6.5	10.4	15.1	< 0.007 (+)
Imipenem-resistant *Pseudomonas aeruginosa *(10,468)	24.0	22.8	23.5	23.8	22.0	24.4	27.0	25.5	No trend
Ciprofloxacin-resistant *Pseudomonas aeruginosa *(11,590)	19.7	18.4	15.6	19.5	17.4	19.3	17.0	16.0	No trend
Imipenem-resistant *Acinetobacter baumannii *(2,014)	1.1	0.7	1.0	2.5	6.3	20.3	9.7	4.5	0.002 (+)
Imipenem-resistant *Klebsiella pneumoniae *(5,732)	0.4	0.2	0.3	0.3	0.0	0.3	0.4	1.1	NA

ICU, intensive care unit; 3GC, third-generation cephalosporin; NA, not applicable because the assumptions of normal distribution are not fulfilled. *P value for the linear regression coefficient; +, increase; -, decrease.

Resistance differed by type of ICU: ciprofloxacin resistance in *P. aeruginosa *was significantly higher in medical and interdisciplinary ICUs than in surgical ICUs. In contrast, vancomycin resistance in *E. faecium *was significantly higher in medical ICUs.

**Figure 3:**
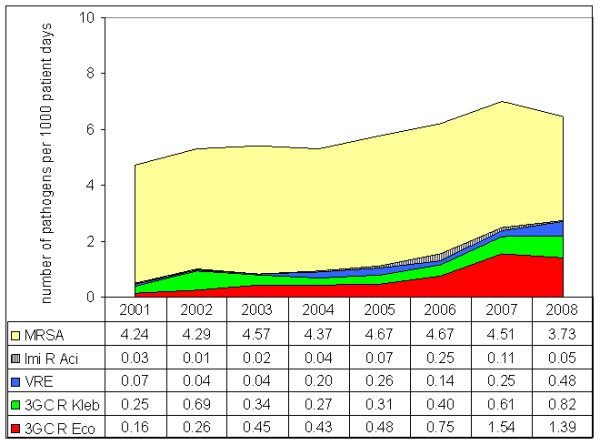
**Change in burden of resistance of multidrug-resistant pathogens from 2001 to 2008**. MRSA, methicillin-resistant *Staphylococcus aureus*; Imi R Aci, imipenem-resistant *Acinetobacter baumannii*; VRE, vancomycin-resistant *Enterococcus faecium*; 3GC R Kleb, third-generation cephalosporin-resistant *Klebsiella pneumoniae*; 3GC R Eco, third-generation cephalosporin-resistant *Escherichia coli*.

It is also possible to demonstrate the different dynamics of resistant pathogens if the RD is used as a parameter for the burden of resistance (Figure 3). Although at 3.73 MRSA/1,000 patient-days MRSA still presented the highest burden of resistant pathogens in 2008, the burden of 3GC-resistant *E. coli *was a remarkable 1.39 ESBL isolates/1,000 patient-days. This means that in 2008 the RD of 3GC-resistant *E. coli *I was equivalent to more than one-third of the overall RD of MRSA. The burden of imipenem-resistant *K. pneumoniae *was still low at 0.05 per 1,000 patient-days in 2008, which corresponds to seven isolates from two separate ICUs.

**Figure 4:**
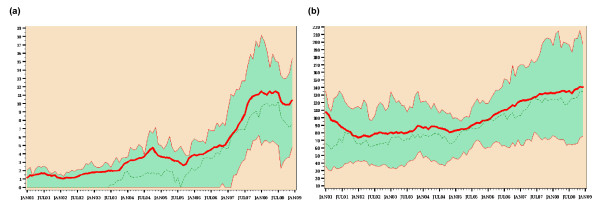
**Third-generation cephalosporin-resistant *Escherichia coli *and carbapenem use from 2001 to 2008**. **(a) **Percentage of third-generation cephalosporin-resistant (3GC) *Escherichia coli*. The pooled mean (solid line) and the median (dotted line) run almost parallel to one another. The sharp increase in 3CG-resistant *E. coli *starts in 2006 and affects almost all intensive care units (ICUs). The interquartile range (shaded area) shows that 50% of all ICUs had resistance proportions between 5 and 15% in 2008. **(b) **Carbapenem use. The pooled mean (solid line) and the median (dotted line) run almost parallel to one another. In most ICUs the increase in carbapenem use also starts in 2006. The interquartile range (shaded area) shows that carbapenem use in 50% of all ICUs ranged between 70 and 190 daily defined doses (DDD)/1,000 patient-days (pd) in 2008.

Figure 4a, b shows the pooled mean and the median of carbapenem use and 3GC resistance in *E. coli*. Resistance started to increase in parallel in 2006, indicating that the increase was not only based on some outlier ICUs but affected almost all ICUs. Indeed, since 2006 just four ICUs have not been confronted with 3GC-resistant *E. coli*. The resistance proportions of 3GC-resistant *E. coli *in the other ICUs ranged from 2 to 24% and the RD from 0.1 to 4.6. The burden of 3GC-resistant *E. coli *even outnumbered the burden of MRSA in seven ICUs.

## Discussion

The three main findings of this study are: that total antibiotic consumption remained stable from 2001 through 2008 with a mean use of 1.2 DDD per patient per ICU-day; that the burden of resistance increased dramatically for 3GC-resistant *E. coli *and *K. pneumoniae *over 8 years, but not for MRSA; and that our data demonstrate the dangerous spiral of spread of resistance and antibiotic use - the increase in 3GC resistance, which indicates a rise in ESBL-producing bacteria, has been followed by a doubling of carbapenem use that, in turn, might now be followed by an increase in imipenem-resistant pathogens.

Antibiotic consumption varied widely by the factor of five, which may partially be explained by differences in patients and ICU characteristics, antibiotic policies or physicians' level of education. Although quantitative data must not be taken as qualitative parameters, the heterogeneity of antibiotic prescriptions might still indicate that antimicrobial use can be improved. We were able to show in three intervention studies that it was indeed possible to sustainably reduce antibiotic consumption in SARI ICUs by shortening the duration of treatment or revising antibiotic prophylaxis [16-18]. Generally, total median antibiotic use of 1,213 DDD/1,000 patient-days concurs with data from 35 ICUs in eight European countries in 2005, with a median of 1,254 DDD/1,000 patient-days (also ranging widely from 348 to 4,992 DDD/1,000 patient-days) [19]. In Swedish ICUs, antibiotic use increased significantly from 1,245 DDD/1,000 patient-days in 1999 to 1,510 DDD/1,000 patient-days in 2003. In these ICUs, antibiotic prescribing was empiric and adequate [20,21]. It can be assumed that antibiotic therapy is also widely empiric in SARI ICUs because the mean ICU stay was only 4.0 days. Empiric antibiotic therapy should be timely and adequate; however, it will normally be broad in the critically ill. The changed resistance situation - that is, the increase in 3GC-resistant *E. coli *- was associated with a doubling in carbapenem use.

The European Antimicrobial Resistance Surveillance System described resistance against 3GC in *E. coli *as the most dynamic expansion of multidrug-resistant pathogens in the entire region [22]. Although in 2008 just under one-half of European countries (14 of 33) reported their resistance levels against 3GC to be under 5%, since 2004 the proportion of 3GC resistance has increased in 19 European countries. In general, a large percentage of ESBL-producing pathogens are now being imported into hospitals and ICUs [23-25]. Known risk factors inside and outside the hospital include use especially of broad-spectrum cephalosporins and quinolones, which, in some ICUs at least, are the workhorses of antibiotic therapy [26-28]. Use of these antibiotic classes might also contribute to the selection of ESBLs, to the persistence of predominant ESBL clones and to the probable dissemination of conjugative plasmids among strains. Limiting administration of these antibiotics to patients in which other therapeutic alternatives according to evidence-based guidelines are not possible is therefore part of many antibiotic stewardship programmes.

A shift toward greater carbapenem usage harbours the risk of greater selection of carbapenem resistance and is associated with permeability mutations in strains already producing ESBLs or other potent β-lactamases [29]. This is already happening, especially in *Klebsiella*. Current reports indicate that *K. pneumoniae *carbapenemases (KPC) are widespread in China, Israel, Greece, South America, and the USA [29,30]. Fortunately, KPC-producing bacteria are still rare in Western Europe and Northern Europe. Although not yet broadly reflected in our data - only two ICUs encountered imipenem-resistant *K. pneumoniae *in 2008 - it is to be expected that such strains will also be increasingly encountered in European ICUs, because it has been shown that high level carbapenem-resistant KPC-producing bacteria may be selected during imipenem and meropenem therapy [31]. In contrast, the increase in 3GC-resistant *E. coli *has now affected most ICUs and is therefore unlikely to be a problem caused by the selection pressure of antibiotic therapy in the individual ICU. If the import into the ICU and the burden of multiresistant pathogens continues to increase, however, adopting appropriate infection control strategies becomes paramount to prevent the transmission to other patients. Besides the basic set of infection control measures, early identification by screening and accommodating patients with multidrug-resistant pathogens in single rooms or cohort isolation is recommended [32].

The spread and emergence of resistance is multifaceted; it is not driven by antibiotic use alone, but is, among other things, also influenced by clonal spread of strains, by resistance mechanisms that might differ by species, the human and environmental reservoir, and by infection control strategies, including screening policies. We hypothesise that these factors at least partly explain why MRSA resistance did not increase over the study period but VRE did, why imipenem-resistant *K. pneumoniae *increased but imipenem-resistant *P. aeruginosa *did not, or why ciprofloxacin-resistant *E. coli *increased yet ciprofloxacin-resistant *P. aeruginosa *did not.

For instance, cumulative German prevalence data from 2007 show that the percentage of ciprofloxacin-resistant *E. coli *was even higher in the outpatient setting than in ICU patients (29.2% vs. 21.9%) [33]. This indicates that quinolone-resistant strains of *E. coli *are imported to ICUs through the massive selective pressure of quinolones prescribed in the outpatient setting in Germany; outpatient antibiotic use accounts for 85% of total antibiotic use [34]. Furthermore, use of quinolones in therapy and prophylaxis, especially in commercial poultry farming, contributes to the emergence of resistant organisms in the human population, especially in pathogens having their reservoir in the gut. In contrast, the natural reservoir of *A. baumannii *is unknown and *A. baumannii *is rarely found on the human skin or in the environment. Higgins and colleagues presented global data on carbapenem-resistant *A. baumannii *suggesting that carbapenem resistance developed after or during the spread of the clonal lineages [35]. They explained that clonal lineages originated worldwide in at least eight distinct loci and then spread to new locations, possibly through patient transfer. If clonal spread is probably responsible for the spread of carbapenem-resistant *A. baumannii*, then *A. baumannii *infections may indicate a serious infection-control problem. Although there was a temporal association between increases in carbapenem use and imipenem resistance in *A. baumannii *in our study, it does not prove a causal relationship.

The present study has several limitations. Whereas the ecological study design can lead to the formation of a hypothesis, ultimately it does not prove a causative relationship - which a patient-based study design is able to establish. Potential confounders such as antimicrobial stewardship interventions (for example, the feedback of the data to the ICUs is a type of intervention) or promotion of hand hygiene could have influenced antibiotic use and antimicrobial resistance in individual ICUs. The number of ICUs participating in SARI increased from 36 to 53, with 47% of the ICUs reporting data over the 8-year period. ICUs that joined recently might therefore have had different antimicrobial usage patterns, as well as a different antimicrobial resistance situation. Outbreaks might have influenced pooled mean resistance data. World Health Organisation DDD do not always correctly reflect the actual prescribed daily dose [36,37]. This inconsistency does not invalidate the systematic approach of the World Health Organisation, and therefore ICUs should use the DDD to make national and international comparisons of their antibiotic use.

## Conclusions

Disturbingly, ICUs have little in reserve to control multidrug resistance among Gram-negative bacteria [38]. We consider five points to be of paramount importance. Firstly, ICUs must ensure proper detection in their laboratories of extended-spectrum β-lactamases among KPC-producing Enterobacteriaceae clinical isolates [39]. Secondly, treatment options for infections with multiresistant Gram-negative bacteria and KPC-producing organisms are limited. Besides tigecycline, therefore, old antibiotics like aminoglycosides, fosfomycin, colistin and rifampicin will have to be re-employed. Thirdly, treatment duration should be as short as is clinically feasible to reduce selection pressure [40]. Fourthly, screening on admission, as already established for MRSA, might be considered to address the increasing import of resistant pathogens into the ICU and to minimise the risk of transmission [24]. Finally, infection control - especially hand hygiene, the purpose of which is to prevent person-to-person spread - is elementary and crucial.

## Key messages

• The burden of resistance increased dramatically for 3GC-resistant *E. coli *and *K. pneumoniae*, which indicates a rise in ESBL-producing bacteria.

• This increase has been followed by a doubling of carbapenem use from 2001 through 2008.

• Greater carbapenem use harbours the risk of greater selection of carbapenem resistance and is associated with permeability mutations in strains already producing ESBLs or KPCs.

• Because treatment options for infections with multiresistant Gram-negative bacteria and KPC-producing organisms are limited, besides tigecycline, old antibiotics such as aminoglycosides, fosfomycin, and colistin with or without rifampicin will have to be re-employed.

• Adopting appropriate infection control strategies becomes paramount to prevent transmission to other patients.

## Abbreviations

AD: antimicrobial usage density; DDD: daily defined doses; ESBL: extended-spectrum β-lactamase; 3GC: third-generation cephalosporin; ICU: intensive care unit; KPC: *Klebsiella pneumoniae *carbapenemase; MRSA: methicillin-resistant *Staphylococcus aureus*; RD: resistance densities; SARI: Surveillance of Antibiotic Use and Bacterial Resistance in Intensive Care Units; VRE: vancomycin-resistant enterococcus.

## Competing interests

The authors declare that they have no competing interests.

## Authors' contributions

All authors have contributed substantially to the submitted work and have read and approved the final manuscript. EM wrote the manuscript, FS analysed the data, BSB collected the data and PG revised the manuscript critically.

## References

[B1] WHO Global Strategy for Containment of Antimicrobial Resistance http://www.who.int/drugresistance/WHO_Global_Strategy_English.pdf

[B2] Livermore DM Bacterial resistance: origins, epidemiology, and impact Clin Infect Dis 2003 36 S11 S23 10.1086/344654 12516026

[B3] Foster KR Grundmann H Do we need to put society first? The potential for tragedy in antimicrobial resistance PLoS Med 2006 3 e29 10.1371/journal.pmed.0030029 16398572 PMC1325265

[B4] Maragakis LL Perencevich EN Cosgrove SE Clinical and economic burden of antimicrobial resistance Expert Rev Anti Infect Ther 2008 6 751 763 10.1586/14787210.6.5.751 18847410

[B5] Bronzwaer SL Cars O Buchholz U Molstad S Goettsch W Veldhuijzen IK Kool JL Sprenger MJ Degener JE A European study on the relationship between antimicrobial use and antimicrobial resistance Emerg Infect Dis 2002 8 278 282 10.3201/eid0803.010192 11927025 PMC2732471

[B6] Jones ME Draghi DC Thornsberry C Karlowsky JA Sahm DF Wenzel RP Emerging resistance among bacterial pathogens in the intensive care unit - a European and North American Surveillance study (2000-2002) Ann Clin Microbiol Antimicrob 2004 3 14 15283864 10.1186/1476-0711-3-14 PMC509280

[B7] Molstad S Erntell M Hanberger H Melander E Norman C Skoog G Lundborg CS Soderstrom A Torell E Cars O Sustained reduction of antibiotic use and low bacterial resistance: 10-year follow-up of the Swedish Strama programme Lancet Infect Dis 2008 8 125 132 10.1016/S1473-3099(08)70017-3 18222163

[B8] Vander Stichele RH Elseviers MM Ferech M Blot S Goossens H Hospital consumption of antibiotics in 15 European countries: results of the ESAC Retrospective Data Collection (1997-2002) J Antimicrob Chemother 2006 58 159 167 10.1093/jac/dkl147 16698845

[B9] Meyer E Gastmeier P Schwab F The burden of multiresistant bacteria in German intensive care units J Antimicrob Chemother 2008 62 1474 1476 10.1093/jac/dkn391 18794158

[B10] Meyer E Jonas D Schwab F Rueden H Gastmeier P Daschner FD Design of a surveillance system of antibiotic use and bacterial resistance in German intensive Care units (SARI) Infection 2003 31 208 215 14562943 10.1007/s15010-003-3201-7

[B11] Meyer E Schwab F Gastmeier P Jonas D Rueden H Daschner FD Methicillin-resistant Staphylococcus aureus in German intensive care units during 2000-2003: data from project SARI (Surveillance of Antimicrobial Use and Antimicrobial Resistance in Intensive Care Units) Infect Control Hosp Epidemiol 2006 27 146 154 10.1086/500619 16465631

[B12] Surveillance der Antibiotika-Anwendung und der bakteriellen Resistenzen auf Intensivstationen http://www.nrz-hygiene.de/sari/sari.htm

[B13] WHO Collaborating Centre for Drug Statistics Methodology ATC/DDD Index http://www.whocc.no/atc_ddd_index/

[B14] Leitlinien und Empfehlungen zur Sicherung von Guter Epidemiologischer Praxis (GEP) http://www.rki.de/cln_169/nn_205212/DE/Content/GBE/EpidemiologischeMethoden/Empfehlungen/empfehlungen__pdf1,templateId=raw,property=publicationFile.pdf/empfehlungen_pdf1.pdf 10893878

[B15] Gesetz zur Verhütung und Bekämpfung von Infektionskrankheiten beim Menschen http://bundesrecht.juris.de/ifsg/__23.html

[B16] Meyer E Buttler J Schneider C Strehl E Schroeren-Boersch B Gastmeier P Ruden H Zentner J Daschner FD Schwab F Modified guidelines impact on antibiotic use and costs: duration of treatment for pneumonia in a neurosurgical ICU is reduced J Antimicrob Chemother 2007 59 1148 1154 10.1093/jac/dkm088 17434880

[B17] Meyer E Lapatschek M Bechtold A Schwarzkopf G Gastmeier P Schwab F Impact of restriction of third generation cephalosporins on the burden of third generation cephalosporin resistant *K. pneumoniae *and *E. coli *in an ICU Intensive Care Med 2009 35 862 870 10.1007/s00134-008-1355-6 19034426

[B18] Meyer E Schwab F Pollitt A Bettolo W Schroeren-Boersch B Trautmann M Impact of a change in antibiotic prophylaxis on total antibiotic use in a surgical intensive care unit Infection 2010 38 19 24 10.1007/s15010-009-9115-2 19904488

[B19] Hanberger H Arman D Gill H Jindrak V Kalenic S Kurcz A Licker M Naaber P Scicluna EA Vanis V Hanberger H Arman D Gill H Jindrak V Kalenic S Kurcz A Licker M Naaber P Scicluna EA Vanis V Walther SM Surveillance of microbial resistance in European Intensive Care Units: a first report from the Care-ICU programme for improved infection control Intensive Care Med 2009 35 91 100 10.1007/s00134-008-1237-y 18670757

[B20] Erlandsson M Burman LG Cars O Gill H Nilsson LE Walther SM Hanberger H Prescription of antibiotic agents in Swedish intensive care units is empiric and precise Scand J Infect Dis 2007 39 63 69 10.1080/00365540600740504 17366015

[B21] Hanberger H Burman LG Cars O Erlandsson M Gill H Nilsson LE Nordlinder D Walther SM Low antibiotic resistance rates in *Staphylococcus aureus*, *Escherichia coli *and *Klebsiella *spp but not in *Enterobacter *spp and *Pseudomonas aeruginosa*: a prospective observational study in 14 Swedish ICUs over a 5-year period Acta Anaesthesiol Scand 2007 51 937 941 10.1111/j.1399-6576.2007.01364.x 17635399

[B22] European Antimicrobial Resistance Surveillance System annual report 2008 http://www.rivm.nl/earss/Images/EARSS%202008_final_tcm61-65020.pdf

[B23] Harris AD McGregor JC Johnson JA Strauss SM Moore AC Standiford HC Hebden JN Morris JG Risk factors for colonization with extended-spectrum β-lactamase-producing bacteria and intensive care unit admission Emerg Infect Dis 2007 13 1144 1149 17953083 10.3201/eid1308.070071 PMC2828082

[B24] Meyer E Serr A Schneider C Utzolino S Kern WV Scholz R Dettenkofer M Should we screen patients for extended-spectrum β-lactamase-producing enterobacteriaceae in intensive care units? Infect Control Hosp Epidemiol 2009 30 103 105 10.1086/592702 19067603

[B25] Rodriguez-Bano J Navarro MD Romero L Martinez-Martinez L Muniain MA Perea EJ Perez-Cano R Pascual A Epidemiology and clinical features of infections caused by extended-spectrum β-lactamase-producing *Escherichia coli *in nonhospitalized patients J Clin Microbiol 2004 42 1089 1094 10.1128/JCM.42.3.1089-1094.2004 15004058 PMC356843

[B26] Arpin C Quentin C Grobost F Cambau E Robert J Dubois V Coulange L Andre C Nationwide survey of extended-spectrum β-lactamase-producing Enterobacteriaceae in the French community setting J Antimicrob Chemother 2009 63 1205 1214 10.1093/jac/dkp108 19329798

[B27] Ben-Ami R Rodriguez-Bano J Arslan H Pitout JD Quentin C Calbo ES Azap OK Arpin C Pascual A Livermore DM Ben-Ami R Rodriguez-Bano J Arslan H Pitout JD Quentin C Calbo ES Azap OK Arpin C Pascual A Livermore DM Garau J Carmeli Y A multinational survey of risk factors for infection with extended-spectrum β-lactamase-producing enterobacteriaceae in nonhospitalized patients Clin Infect Dis 2009 49 682 690 10.1086/604713 19622043

[B28] Kaier K Frank U Hagist C Conrad A Meyer E The impact of antimicrobial drug consumption and alcohol-based hand rub use on the emergence and spread of extended-spectrum beta-lactamase-producing strains: a time-series analysis J Antimicrob Chemother 2009 63 609 614 10.1093/jac/dkn534 19151036

[B29] Nordmann P Cuzon G Naas T The real threat of *Klebsiella pneumoniae *carbapenemase-producing bacteria Lancet Infect Dis 2009 9 228 236 10.1016/S1473-3099(09)70054-4 19324295

[B30] Kitchel B Sundin DR Patel JB Regional dissemination of KPC-producing *Klebsiella pneumoniae* Antimicrob Agents Chemother 2009 53 4511 4513 10.1128/AAC.00784-09 19687250 PMC2764151

[B31] Hong T Moland ES Abdalhamid B Hanson ND Wang J Sloan C Fabian D Farajallah A Levine J Thomson KS *Escherichia coli*: development of carbapenem resistance during therapy Clin Infect Dis 2005 40 e84 e86 10.1086/429822 15844056

[B32] Siegel JD Rhinehart E Jackson M Chiarello L Management of multidrug-resistant organisms in health care settings, 2006 Am J Infect Control 2007 35 S165 S193 10.1016/j.ajic.2007.10.006 18068814

[B33] Kresken M Hafner D Schmitz FJ Wichelhaus TA Resistenzsituation bei klinisch wichtigen Infektionserregern gegenüber Antibiotika in Deutschland und im mitteleurop,,ischen Raum http://www.p-e-g.org/ag_resistenz/main.htm

[B34] GERMAP 2008 Antibiotika-Resistenz und -Verbrauch http://www.bvl.bund.de/cln_007/DE/08__PresseInfothek/00__doks__downloads/Germap__2008,templateId=raw,property=publicationFile.pdf/Germap_2008.pdf

[B35] Higgins PG DC Hackel M Seifert H Global spread of carbapenem-resistant *Acinetobacter baumannii* J Antimicrob Chemother 2010 65 233 238 10.1093/jac/dkp428 19996144

[B36] de With K Bestehorn H Steib-Bauert M Kern WV Comparison of defined versus recommended versus prescribed daily doses for measuring hospital antibiotic consumption Infection 2009 37 349 352 10.1007/s15010-008-8138-4 19277464

[B37] Muller A Monnet DL Talon D Henon T Bertrand X Discrepancies between prescribed daily doses and WHO defined daily doses of antibacterials at a university hospital Br J Clin Pharmacol 2006 61 585 591 10.1111/j.1365-2125.2006.02605.x 16669851 PMC1885049

[B38] Livermore DM Has the era of untreatable infections arrived? J Antimicrob Chemother 2009 64 Suppl 1 i29 i36 10.1093/jac/dkp255 19675016

[B39] Tsakris A Poulou A Themeli-Digalaki K Voulgari E Pittaras T Sofianou D Pournaras S Petropoulou D Use of boronic acid disk tests to detect extended-spectrum β-lactamases in KPC carbapenemase-possessing enterobacteriaceae clinical isolates J Clin Microbiol 2009 47 3420 3426 10.1128/JCM.01314-09 19726597 PMC2772593

[B40] Chastre J Wolff M Fagon JY Chevret S Thomas F Wermert D Clementi E Gonzalez J Jusserand D Asfar P Chastre J Wolff M Fagon JY Chevret S Thomas F Wermert D Clementi E Gonzalez J Jusserand D Asfar P Perrin D Fieux F Aubas S Comparison of 8 vs 15 days of antibiotic therapy for ventilator-associated pneumonia in adults: a randomized trial JAMA 2003 290 2588 2598 10.1001/jama.290.19.2588 14625336

